# Supplementary material for: Northeast Pacific warm blobs sustained via extratropical atmospheric teleconnections
Source: Nat Commun. 2024 Apr 2;15:2832. doi: 10.1038/s41467-024-47032-x (PMC10987500; doi:10.1038/s41467-024-47032-x)
Supplement: Supplementary file 1 — Supplementary Information [file 41467_2024_47032_MOESM1_ESM.pdf]

Supplementary Table 1: List of NEP warm blob events. Start time, end time, duration, peak time, peak intensity, and return period of each winter-peak warm blob event used in this study.

start time	end time	duration (in month)	peak time	peak intensity (in std.)	return period (in year)
DEC 1956	MAY 1957	6	FEB 1957	2.25	12.6
AUG 1957	DEC 1957	5	NOV 1957	1.61	6.2
OCT 1961	SEP 1962	12	FEB 1962	2.12	10.9
JAN 1963	SEP 1963	9	FEB 1963	2.54	17.4
SEP 1985	MAR 1986	7	NOV 1985	1.74	7.2
SEP 1989	JAN 1990	5	DEC 1989	1.47	5.4
DEC 1990	JUL 1991	8	JAN 1991	2.04	9.9
OCT 1993	MAR 1994	6	NOV 1993	1.74	7.1
NOV 2004	MAY 2005	7	JAN 2005	1.79	7.6
NOV 2013	SEP 2014	11	JAN 2014	3.37	45.3
JAN 2015	NOV 2015	11	MAR 2015	1.91	8.6
MAY 2019	DEC 2020	20	NOV 2019	2.70	20.8
MAY 2019	DEC 2020	20	NOV 2020	2.16	11.4

Supplementary Table 2: List of CAM5 experiments.

Experiments	Prescribed SST forcing	Integration
CTRL	Monthly global climatological SST over 1981–2010	25 years
Exp_Med	Add composite SST anomalies during the warm blobs in the Mediterranean to climatological SST	25 years
Exp_Med_rain	Add SST anomalies averaged in 2013 and 2019 in the Mediterranean to climatological SST	25 years
Exp_NAtl	Add composite SST anomalies during the warm blobs in the NATL to climatological SST	25 years
Exp_NAtl_NAO	Add SST anomalies in 1993 in the NATL to climatological SST	25 years
EXP_TP-only	Add composite SST anomalies during the warm blobs in the tropical Pacific to climatological SST	25 years
EXP_TP+NAtl	Add composite SST anomalies during the warm blobs in the tropical Pacific and NATL to climatological SST	25 years
EXP_TP+Med	Add composite SST anomalies during the warm blobs in the tropical Pacific and Mediterranean to climatological SST	25 years

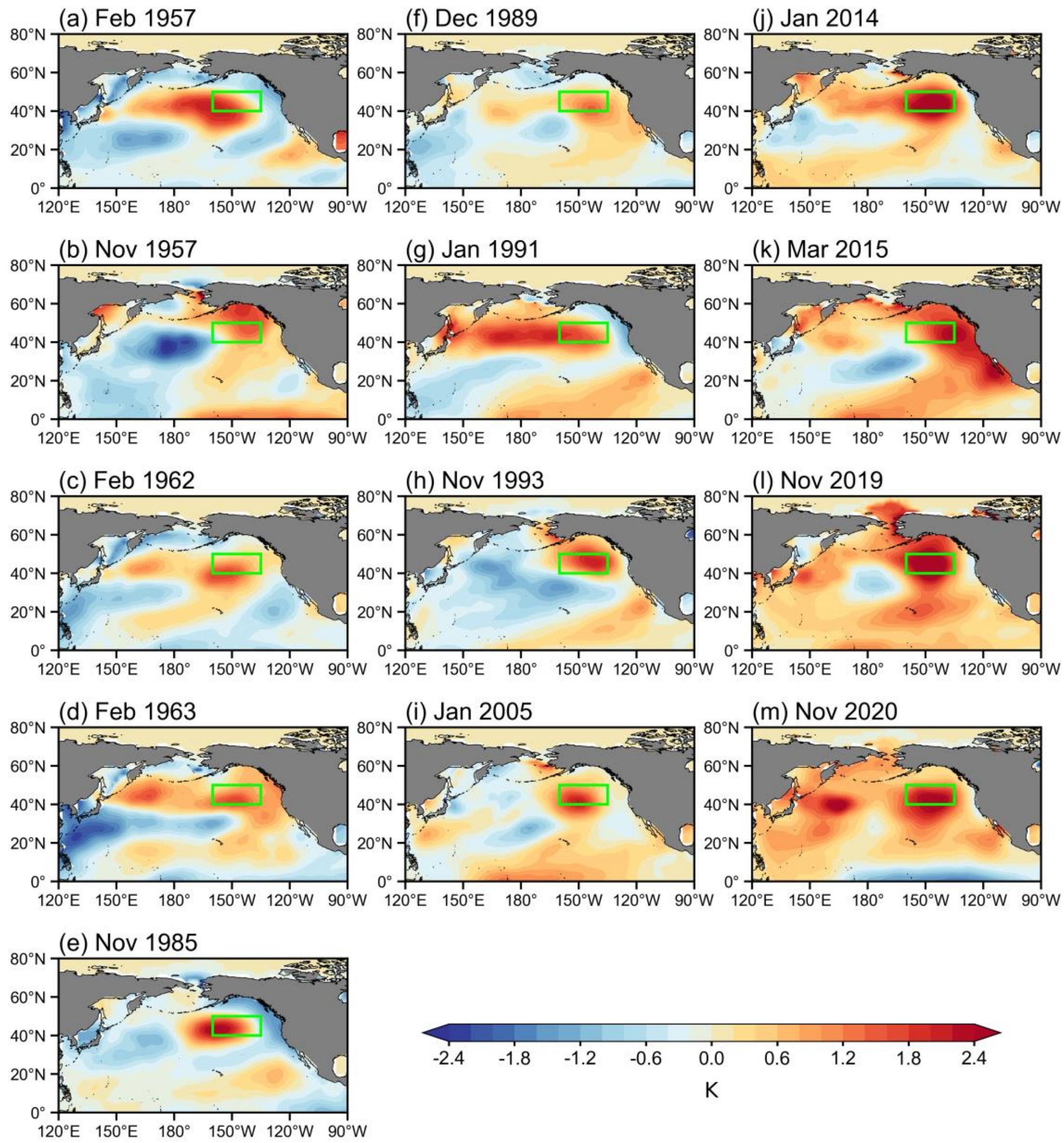

Supplementary Fig. 1 SST anomalies (shading, in °C) at the peak month of each warm blob event. Green boxes represent the study area of the warm blob.

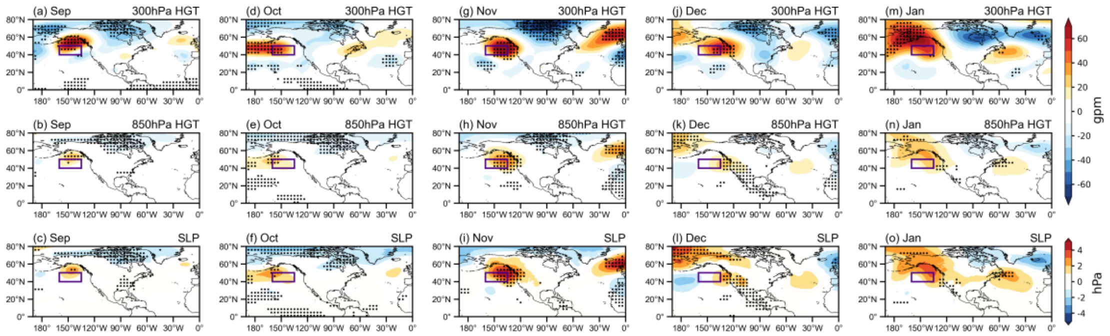

Supplementary Fig. 2 (upper row; a–d) As in Fig. 1, but for 300 hPa from September to following January. (middle row; f–j) As in upper row, but for 850 hPa. (bottom row; k–o) As in upper row, but for SLP anomalies (shading, in hPa). Purple boxes represent the study area of the warm blob. Stippling indicates geopotential height or SLP exceeding a 0.1 significance level based on the two-tailed Student’s *t*-test.

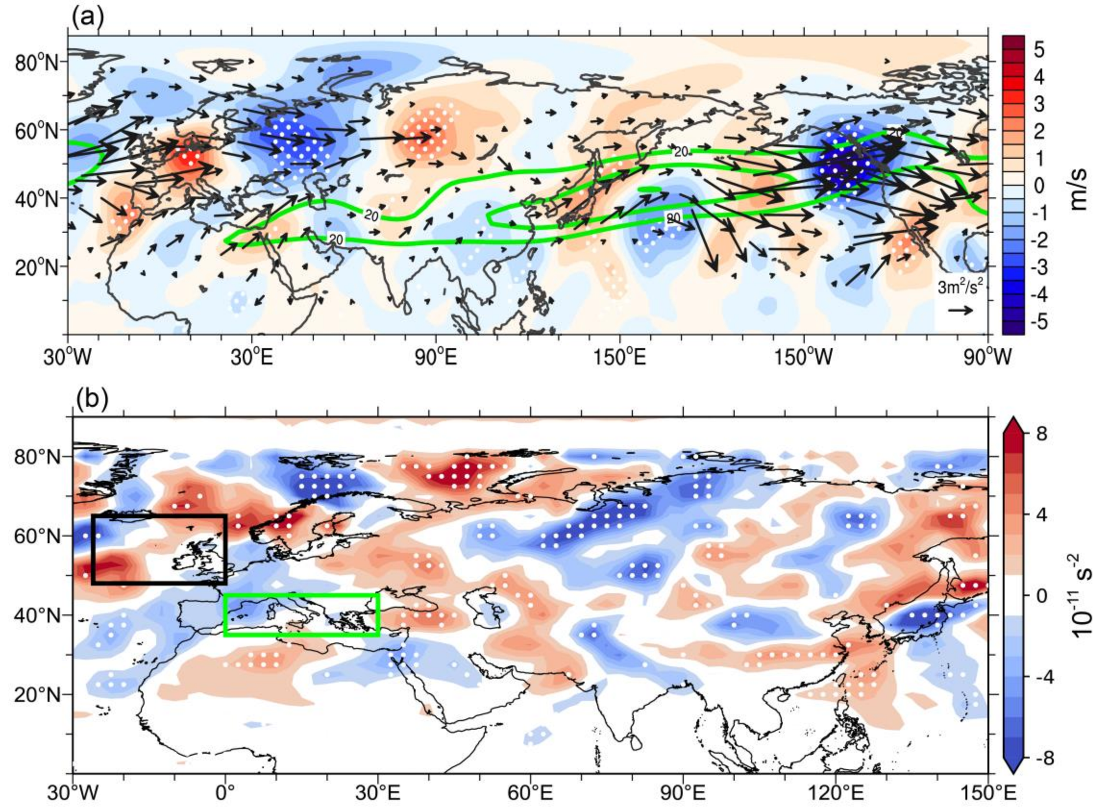

Supplementary Fig. 3 As in Fig. 2, but for October.

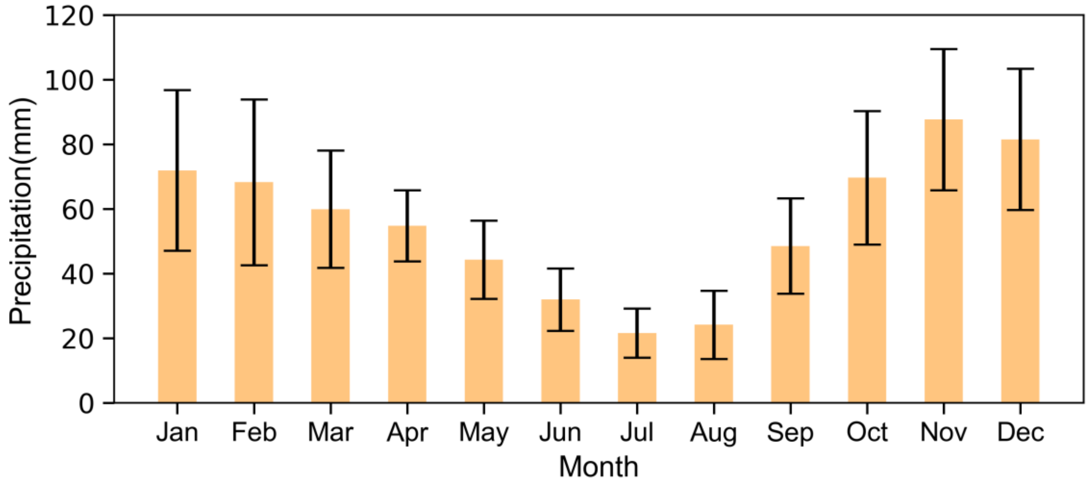

Supplementary Fig. 4 Monthly rainfall climatology (bars, in mm) and standard deviations (errorbars, in mm) over the Mediterranean (box in Fig. 2b).

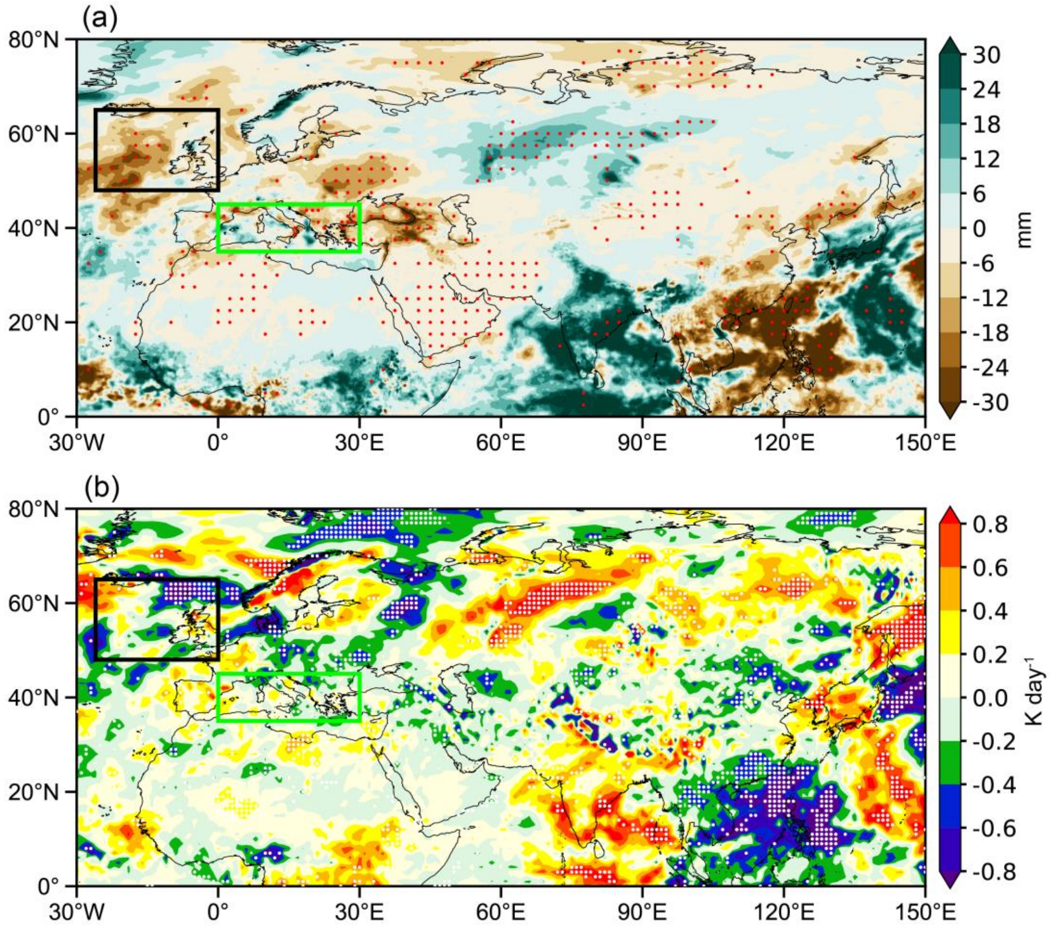

Supplementary Fig. 5 As in Fig. 3, but for October.

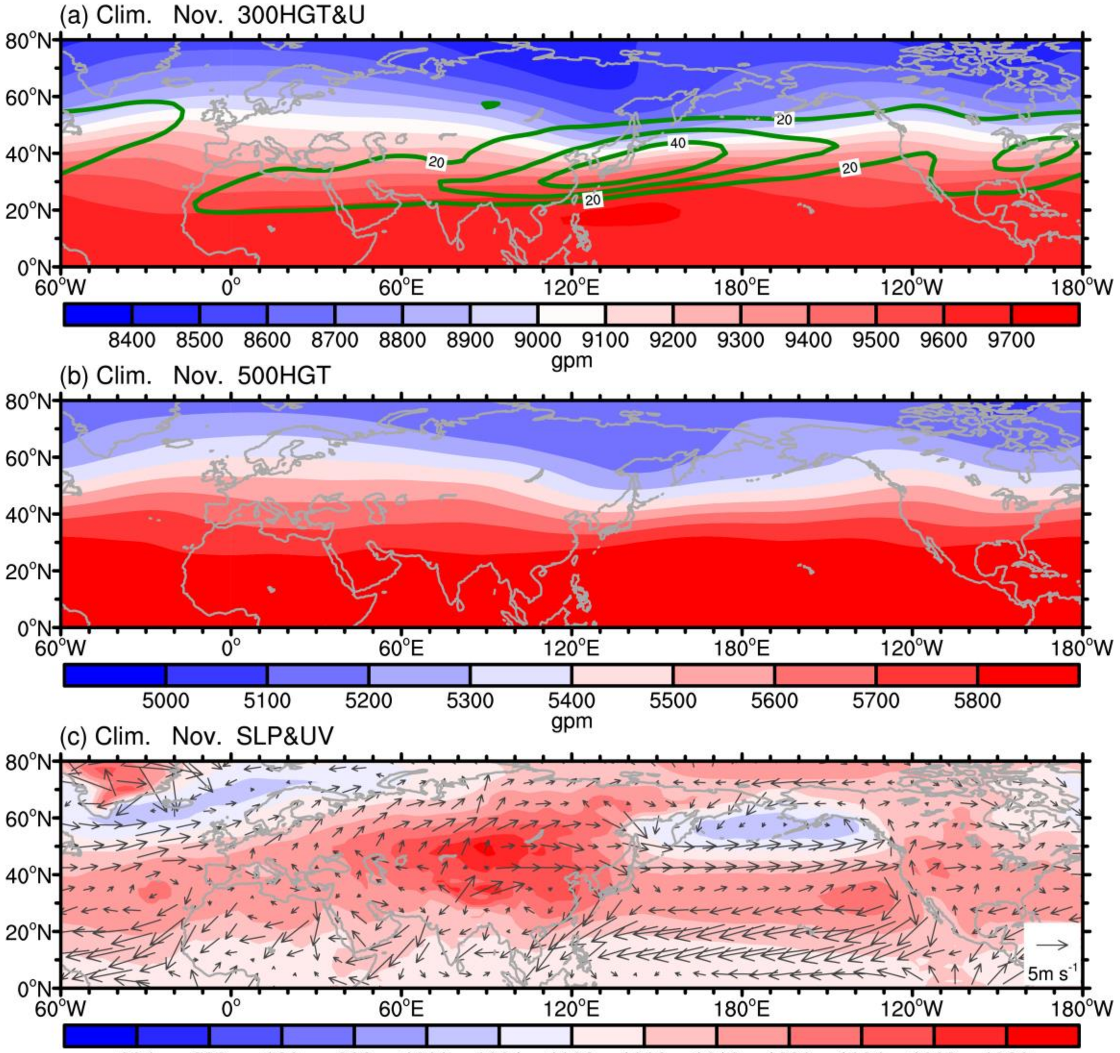

Supplementary Fig. 6 Background climatology of (a) geopotential height (shading, in gpm) and zonal wind (contour, in m/s) at 300 hPa, (b) geopotential height (shading, in gpm) at 500 hPa, and (c) SLP (shading, in hPa) and surface winds (vector, in m/s) in November given in LBM.

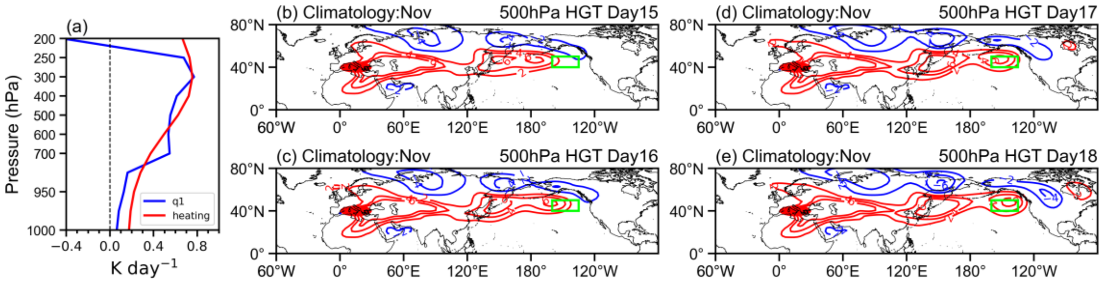

Supplementary Fig. 7 Response of geopotential height anomalies (contours, 2 gpm interval) at 500 hPa to (a) anomalous heating in the LBM on days 15 (b), 16 (c), 17 (d), and 18 (e), respectively. Solid red ellipsoids denote the imposed heat forcing over the Mediterranean. Green boxes represent the warm blob study area.

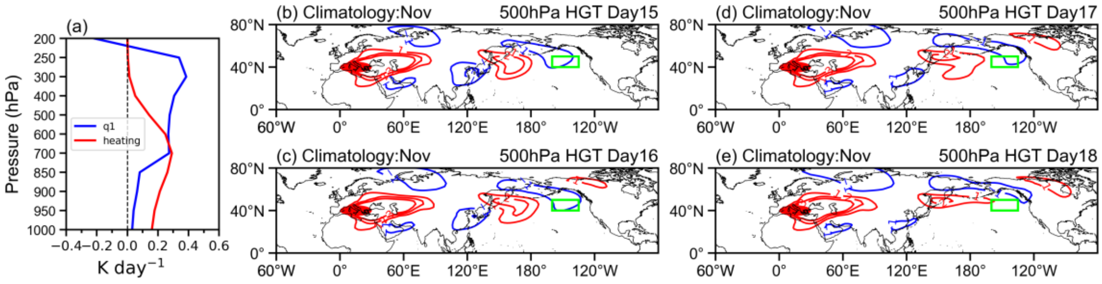

Supplementary Fig. 8 As in Supplementary Fig. 7, but imposing the maximum heating at 700 hPa.

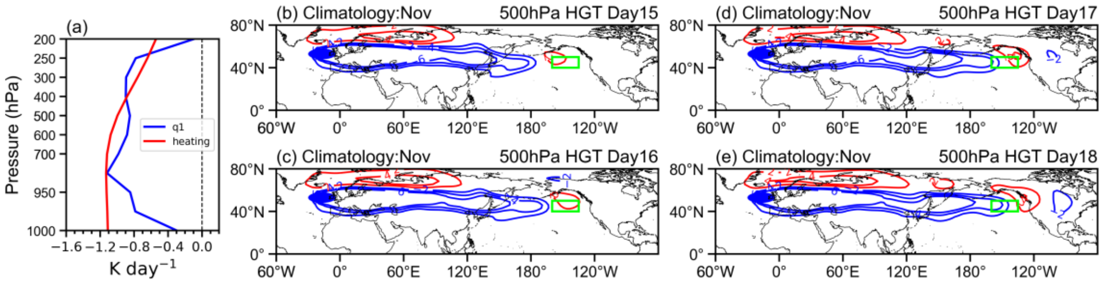

Supplementary Fig. 9 As in Supplementary Fig. 7, but for NATL cooling experiment.

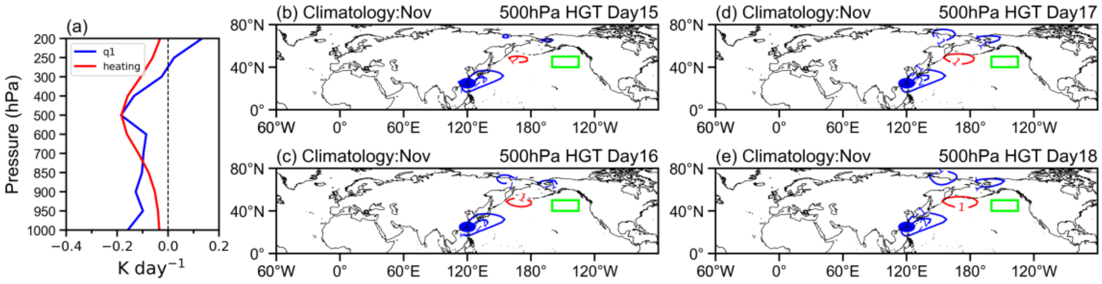

Supplementary Fig. 10 As in Supplementary Fig. 7, but imposing anomalous cooling above southern China and surrounding seas.

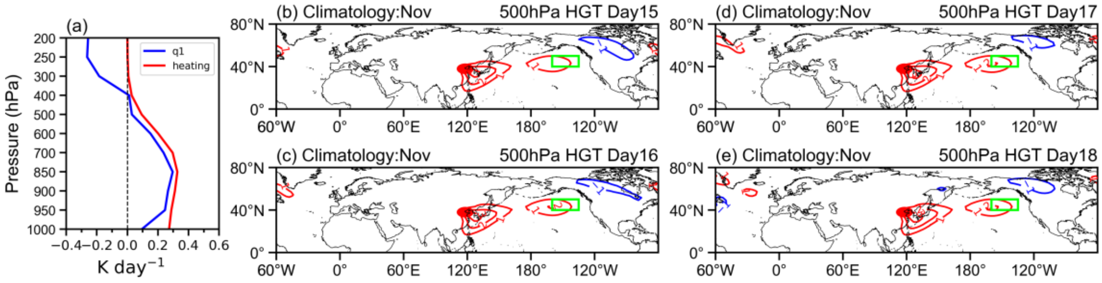

Supplementary Fig. 11 As in Supplementary Fig. 7, but imposing anomalous heating above northern China and surrounding seas.

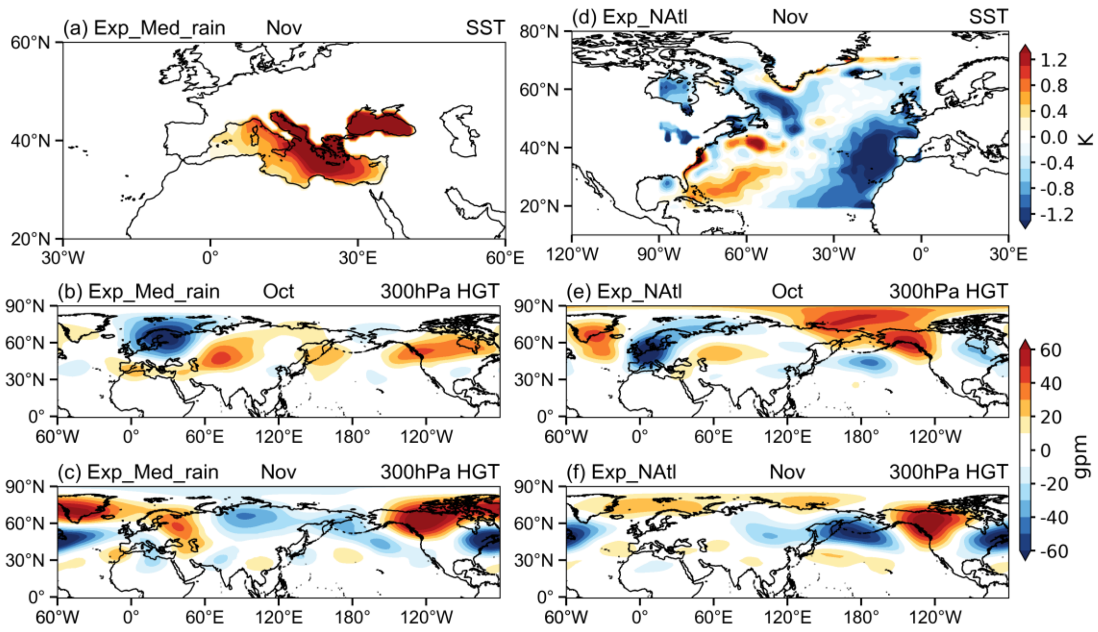

Supplementary Fig. 12 As in Fig. 5, but for Exp_Med_rain run (left column) and Exp_NAtl_NAO run (right column), respectively.

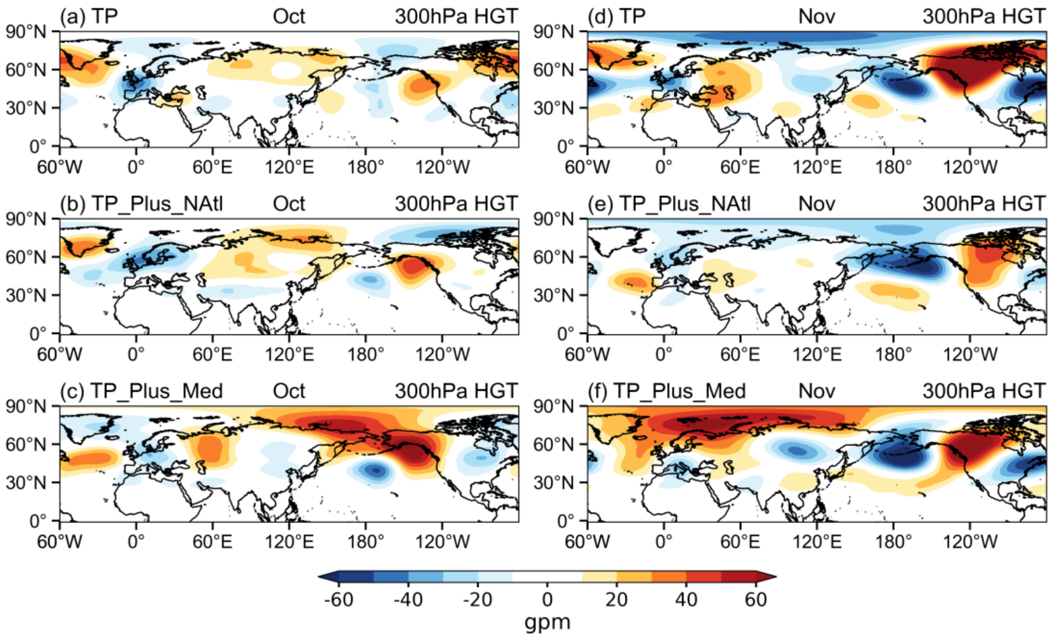

Supplementary Fig. 13 (a,b,c) As in Fig. 5b, but for EXP_TP-only run, EXP_TP+NAtl run, and EXP_TP+Med run, respectively. (d,e,f) As in (a,b,c), but for November.
